# Clinical observation and finite element analysis of cannulated screw internal fixation in the treatment of femoral neck fracture based on different reduction quality

**DOI:** 10.1186/s13018-021-02580-6

**Published:** 2021-07-13

**Authors:** Gan Zhao, Ming Liu, Bin Li, Haizhong Sun, Biaofang Wei

**Affiliations:** 1 Guangzhou University of Chinese Medicine, Guangzhou, 510405 Guangdong China; 2 Department of Sports Medicine, Linyi Traditional Chinese Medicine Hospital, Linyi, 276000 Shandong China; 3 Department of Pain, Linyi People’s Hospital, Linyi, 276000 Shandong Province China; 4 Department of Orthopedic, Linyi People’s Hospital, Linyi, 276000 Shandong Province China

**Keywords:** Femoral neck fracture, Quality of reduction, Cannulated screws, Efficacy, Finite element

## Abstract

**Objective:**

Femoral neck fracture is one of the most common bone types. The effect of reduction quality on hip joint function and complications after screw internal fixation is not fully understood. To investigate the clinical efficacy and mechanical mechanism of positive buttress, anatomical reduction, and negative buttress in the treatment of femoral neck fracture after cannulated screw fixation.

**Methods:**

Retrospective analysis of patients with femoral neck fracture treated with three cannulated screws internal fixation in our hospital from January 2013 to December 2018. According to the quality of fracture reduction, the patients were divided into positive buttress group, anatomical reduction group, and negative buttress group. Basic information such as injury mechanism, time from injury to surgery, Garden classification and Pauwels classification was collected, Harris scores were performed at 3 months, 6 months, and 12 months after surgery, and postoperative complications (femoral head necrosis, femoral neck shortening, and femoral neck nonunion) were collected. At the same time, three groups of finite element models with different reduction quality were established for stress analysis, their stress clouds were observed and the average displacement and stress of the three groups of models were compared. P < 0.05 was used to represent a statistically significant difference.

**Results:**

A total of 225 cases of unilateral femoral neck fractures were included and followed up for an average of 4.12 ± 0.69 years. There was no significant difference in age, gender, side, injury mechanism, time from injury to surgery, BMI, Garden classification, Pauwels classification, and follow-up time among the three groups (P > 0.05). However, there was significant difference in Harris score at 6 and 12 months after operation among the three groups (P < 0.05), which was higher in the positive buttress group and anatomical reduction group than in the negative buttress group. In addition, the incidence of osteonecrosis of the femoral head in the negative buttress group (32.2%) was greater than that in the anatomical reduction group (13.4%) and the positive buttress group (5.4%) (P < 0.05). In addition, the incidence of femoral neck nonunion and femoral neck shortening in the negative buttress group was also higher than that in the anatomical reduction positive buttress group (P < 0.05). The finite element results showed that the stress and fracture end displacement in the negative buttress group were greater than those in the positive buttress group (P < 0.05).

**Conclusion:**

Both positive buttress and anatomical reduction in the treatment of femoral neck fracture with cannulated screw internal fixation can obtain better clinical effect and lower postoperative complications. Positive brace support and anatomic reduction can limit the restoration of femoral stress conduction. Therefore, it is not necessary to pursue anatomical reduction too deliberately during surgery, while negative buttress reduction should be avoided.

## Introduction

Femoral neck fracture is one of the most common fracture types, accounting for approximately 54% of fractures of the entire hip [1]. With the increasing aging of the population, the incidence of femoral neck fractures is increasing year by year [2, 3]. Moreover, due to the rapid development of social economy, high-energy injuries caused by car accidents and falls from height are increasing, and the incidence of femoral neck fractures in young and middle-aged people is increasing year by year [4]. Young and middle-aged patients require joint function activities, so it is necessary to maintain the shape and function of the original joint through reduction as much as possible [5, 6]. One study showed that the incidence of postoperative femoral head necrosis was 16% [7]. At present, there is still a lack of effective methods to prevent complications such as femoral head necrosis after femoral neck fracture surgery.

It remains a challenge for the treatment of femoral neck fractures in young adults, especially Pauwels type III subcapital fractures [8]. The principle of treatment for such injuries is early anatomical reduction and internal fixation with adequate preservation of the blood supply to the femoral head [9]. Many studies have repeatedly emphasized that anatomical reduction is the key to smooth fracture healing [10, 11]. However, in practice, complete anatomical reduction is difficult to achieve, especially with closed reduction methods. In addition, anatomical reduction has the potential to increase operative time and surgical trauma, thus affecting later fracture healing [12]. In 2013, Gotfried [13] proposed a new reduction method to introduce the concepts of “positive buttress” and “negative buttress.” It was preliminarily found that positive buttress could reduce the incidence of postoperative complications, and this method was easy to operate and economical and practical. At present, an article has conducted statistical studies on the differences in the clinical efficacy of “positive buttress” and “negative buttress,” but the number of included cases is small, and none of them has further analyzed the biomechanical mechanism behind it [14]. Finite element analysis is a commonly used research method at present. The advantages of 3D Simulation (FEA) in other applications in orthopedics like tumor bone [15] and thermal necrosis [16]. Therefore, we speculated that the positive buttress reduction method after femoral neck fracture surgery can effectively promote fracture healing and reduce complications. This study is divided into two parts: “positive buttress” clinical efficacy analysis and finite element mechanical analysis. The purpose of this study is to investigate the effect of “anatomical reduction,” “positive buttress,” and “negative buttress” reduction on the incidence rate of complications and clinical efficacy of femoral neck fracture. The mechanical stability of the three reduction results was also explored by finite element analysis in order to provide a reference for clinical treatment of femoral neck fracture surgical reduction.

## Materials and methods

### General information

A retrospective analysis of patients with femoral neck fractures treated with internal fixation with three cannulated screws at Linyi People’s Hospital from January 2013 to December 2018 was performed. Inclusion criteria: (1) Aged > 18 to < 60 years old, (2) clinical and imaging diagnosis of unilateral femoral neck fracture [10], (3) the first three cannulated screw internal fixation treatment, (4) the patient’s medical records and imaging data are complete, agree to cooperate with the follow-up. Exclusion criteria: (1) Pathological fractures, old fractures, and fractures at other sites, (2) receiving non-three cannulated screw internal fixation, (3) suffering from severe cardiovascular, respiratory, and other diseases cannot participate in the operation, (4) patients with incomplete clinical and imaging data. All patients and their families gave informed consent to the treatment protocol, which was approved by the hospital ethics committee No: Y[2019]108.

### Surgical method

All patients were completed by the same surgical team. After the anesthesia (general anesthesia) was significantly effective, routine disinfection and draping were performed; traction bed was used for traction, closed reduction was performed under C-arm machine fluoroscopy, a guide wire was implanted in an inverted triangle parallel calcar about 2 cm distal to the greater trochanter, the skin was incised at the bottom of the guide wire, the tissue was separated until the periosteum, and then three hollow screws arranged in an inverted triangle were inserted after turning the hole and measuring the depth. Postoperative routine antibiotics to prevent infection, get up on the second day after surgery for non-weight-bearing muscle exercise; bed rest for 2-3 months, followed by crutches partial weight-bearing walking for 3 months, postoperative regular review of X and MRI to assess fracture healing and complications.

### Grouping

They were grouped according to the quality of postoperative X-ray fracture reduction. Anatomic reduction group: the alignment between the inner and lower edges of the proximal fracture end and the inner and upper edges of the distal fracture end was neat without displacement; positive buttress group: the inner and lower edges of the distal fracture end protruded medially to the inner and upper edges of the proximal femoral neck fracture end; negative buttress group: the inner and lower edges of the proximal fracture end protruded medially to the inner and lower edges of the distal femoral neck fracture end [13].

### Finite element analysis

**Fig. 1:**
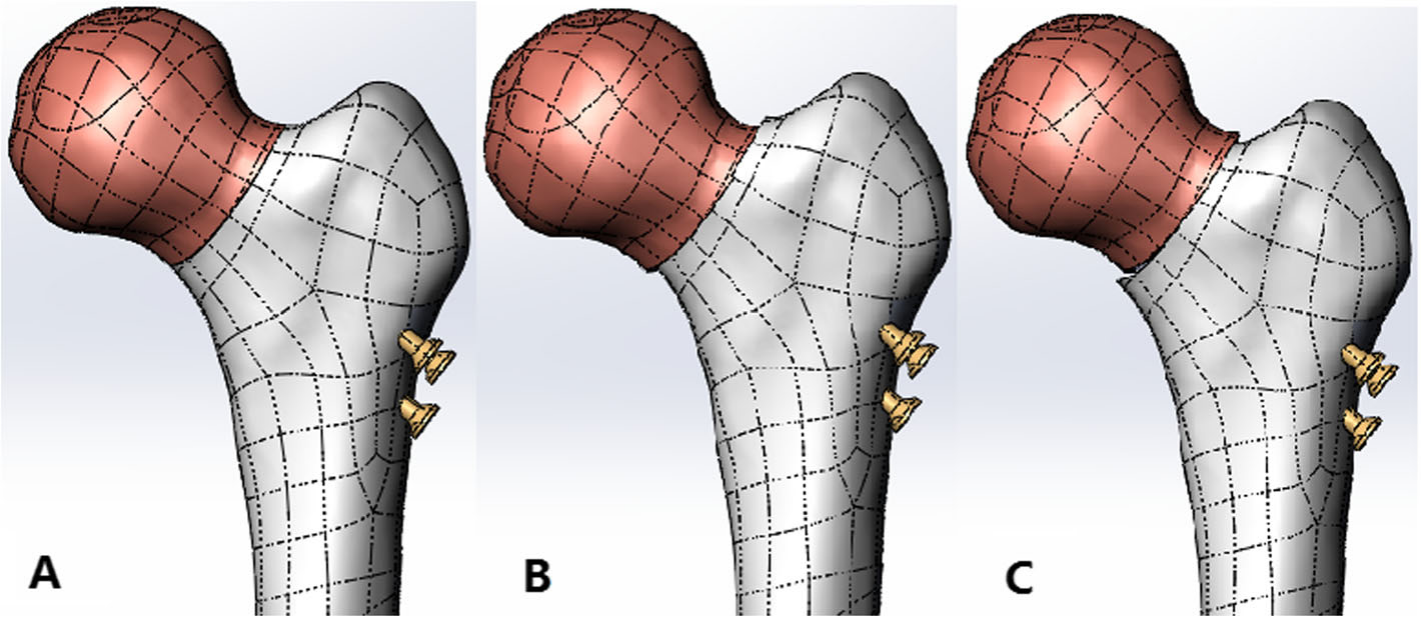
Finite element model: **A** anatomical reduction group, **B** negative buttress group, **C** positive buttress group

A 30-year-old healthy male (height, 170 cm; weight, 65 kg) was selected. The CT data of femoral neck was obtained. The slice thickness was 0.5 mm, the slice distance was 5 mm, the resolution of each slice was 1024 × 1024 pixels. The generated images were saved in DICOM format. Mimics software and the Geomagic-Studio11 software were used for femoral modeling. A femoral neck fracture with a Pauwels angle of 50° was constructed by the Solidworks software, and screw internal fixation was constructed. The reduction was divided into three groups: anatomical reduction group and negative buttress positive buttress group. Mechanical analysis was then performed by Abaqus 6.14, and material properties were assigned: cortical elastic modulus 15100 MPa, Poisson’s ratio 0.3; cancellous elastic modulus 44457 MPa, Poisson’s ratio 0.22; and screw elastic modulus 20600 MPa, Poisson’s ratio 0.3 [17]. The distal femur was fixed and three times the body weight was given above the femoral head. Ten points at the top of the fracture in the three groups of models were taken to measure their displacement; and 10 corresponding stress points were taken from the distance from the femur to record their stress values (Fig. 1).

### Primary outcome measures

The postoperative follow-up femoral neck shortening (using the exposed screw measurement method [10], that is, the neck shortening length = the measured value of the exposed length of the screw rod × the actual thickness of the cannulated screw cap/the measured value of the thickness of the screw cap) was compared among the three groups, and the mild, moderate, and severe degrees (mild, 0-5 mm; moderate, 5-10 mm; and severe, greater than 10 mm), the incidence of nonunion, the incidence of avascular necrosis of the femoral head, the postoperative hip Harris score, and the average displacement and stress of the femoral neck model were classified according to the degree of shortening.

### Statistical analysis

Expressed as‾x ± s, the differences between the groups were compared by one-way analysis of variance, and the SNK method was used for pairwise comparison. Postoperative complications were expressed in % and chi-square tests were performed. Statistical analysis was performed using the SPSS 21.0 software, and P < 0.05 was considered statistically significant.

## Results

### General information

A total of 225 unilateral femoral neck fractures were included, and 3 patients were lost to follow-up, with a mean follow-up of 4.12 ± 0.69 years. There was no significant difference in age, gender, side, mechanism of injury, time from injury to surgery, BMI, Garden classification, Pauwels classification, and follow-up time among the three groups (P > 0.05), and the studies were comparable (Table 1).

**Table 1:** Clinical characteristics of patients in the three groups

Items	Groups			*P* value
	Anatomical reduction group (*n* = 82)	Negative buttress group (*n* = 62)	Positive buttress group (*n* = 78)	
Age (years)	42.6 ± 4.33	42.1 ± 4.47	42.3 ± 4.17	0.784
Gender (%)				
Male	48 (58.5)	36 (58.1)	48 (61.5)	0.896
Female	34 (41.5)	26 (41.9)	30 (38.5)	
Mechanism of injury (%)				
High-energy trauma	50 (61.0)	39 (62.9)	49 (62.8)	0.962
Low-energy trauma	32 (39.0)	23 (37.1)	29 (37.2)	
Side of fracture				
Left	42 (51.2)	30 (48.4)	38 (48.7)	0.929
Right	40 (48.8)	32 (51.6)	40 (51.3)	
Time to surgery (days)	1.80 ± 0.49	1.79 ± 0.50	1.84 ± 0.48	0.849
BMI	23.9 ± 2.23	23.6 ± 2.45	23.7 ± 2.53	0.779
Garden classification				
I	4 (4.9)	2 (3.2)	3 (3.8)	0.980
II	13 (15.9)	11 (17.7)	14 (17.9)	
III	35 (42.7)	30 (48.3)	33 (42.3)	
IV	30 (36.5)	19 (25.8)	28 (36.0)	
Pauwels classification				
I	36 (43.9)	29 (46.8)	34 (43.6)	0.618
II	24 (29.3)	22 (35.5)	22 (28.2)	
III	22 (26.8)	11 (17.7)	21 (26.9)	
Follow-up duration (years)	4.12 ± 0.69	4.11 ± 0.65	4.13 ± 0.58	0.999

### Comparison of postoperative Harris scores among three groups

**Fig. 2:**
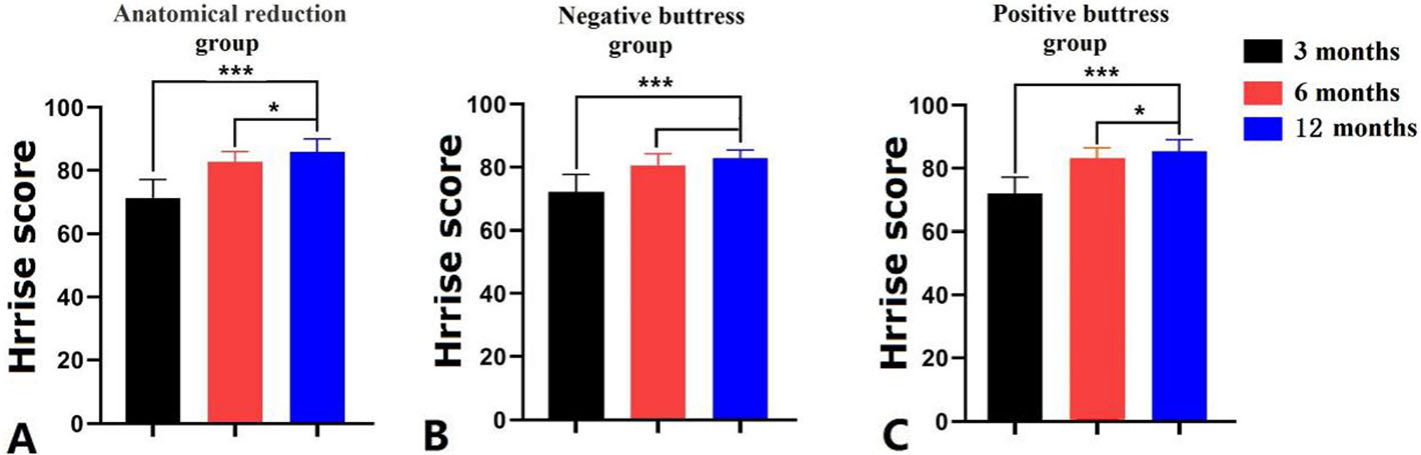
Comparison of postoperative Harris scores among three groups (*P < 0.05; ***P < 0.001)

Harris scores were measured at 3 months, 6 months, and 12 months after screw internal fixation, which increased gradually in the anatomical reduction positive buttress group, and the difference had statistical significance (P < 0.05, Fig. 2). There was no significant difference in Harris score 3 months after operation among the three groups (P > 0.05). However, the difference in the Harris score 6 months after operation among the three groups had statistical significance (P < 0.05). The average Harris score of the negative buttress group was 80.6 ± 3.66 points, which was significantly lower than that of the anatomical reduction group (82.7 ± 3.55) and the positive buttress group (83.1 ± 3.40). There was no statistical difference between the anatomical reduction positive buttress group and the other groups (P > 0.05). The Harris score at 12 months after operation in the three groups was the lowest in the negative buttress group, and the difference had statistical significance (P < 0.05, Table 2, Fig. 2).

**Table 2:** Comparison of postoperative Harris scores among three groups

Groups	3 months	6 months	12 months
Anatomical reduction group (*n* = 82)	71.3 ± 5.80	82.7 ± 3.55*	85.9 ± 4.14*^#^
Negative buttress group (*n* = 62)	72.1 ± 5.59	80.6 ± 3.66*	81.9 ± 2.56*
Positive buttress group (*n* = 78)	72.0 ± 5.24	83.1 ± 3.40*	85.4 ± 3.74*^#^
*P* value	0.601	0.041	< 0.001

*Compared with the Harris score of the same group at 3 months after surgery, the difference was statistically significant (P < 0.05)

^#^Compared with the Harris score of the same group at 6 months after surgery, the difference was statistically significant

### Comparison of the incidence of femoral head necrosis, femoral neck nonunion, and shortening among three groups

There was significant difference in the incidence rate of osteonecrosis of the femoral head among the three groups (P < 0.05), in which the incidence rate of osteonecrosis of the femoral head in the negative buttress group (32.2%) was greater than that in the anatomical reduction group (13.4%) and positive buttress group (5.4%). In addition, the incidence rate of femoral neck nonunion in the negative buttress group (12.9%) was also higher than that in the anatomical reduction group (3.7%) and positive buttress group (2.6%), and the difference had statistical significance (P < 0.05). Femoral neck shortening was most likely to occur in the negative buttress group (27.4%), and was lower than 10% in the other two groups, and the difference was statistically significant (P < 0.05, Table 3).

**Table 3:** Comparison of the incidence of femoral head necrosis, femoral neck nonunion, and shortening among the three groups

Groups	Femoral head necrosis (%)		Femoral neck nonunion (%)		Femoral neck shortening (%)		
	Yes	No	Yes	No	Mild	Moderate	severe
Anatomical reduction group (*n* = 82)	11 (13.4)	71 (86.6)	3 (3.7)	79 (96.3)	3	2	1
Negative buttress group (*n* = 62)	20 (32.2)	42 (67.7)	8 (12.9)	54 (87.1)	8	6	3
Positive buttress group (*n* = 78)	12 (5.4)	66 (84.6)	2 (2.6)	76 (97.4)	2	2	2
*P* value	0.006		0.02		< 0.001		

### Comparison of stress and displacement among three groups of models

**Fig. 3:**
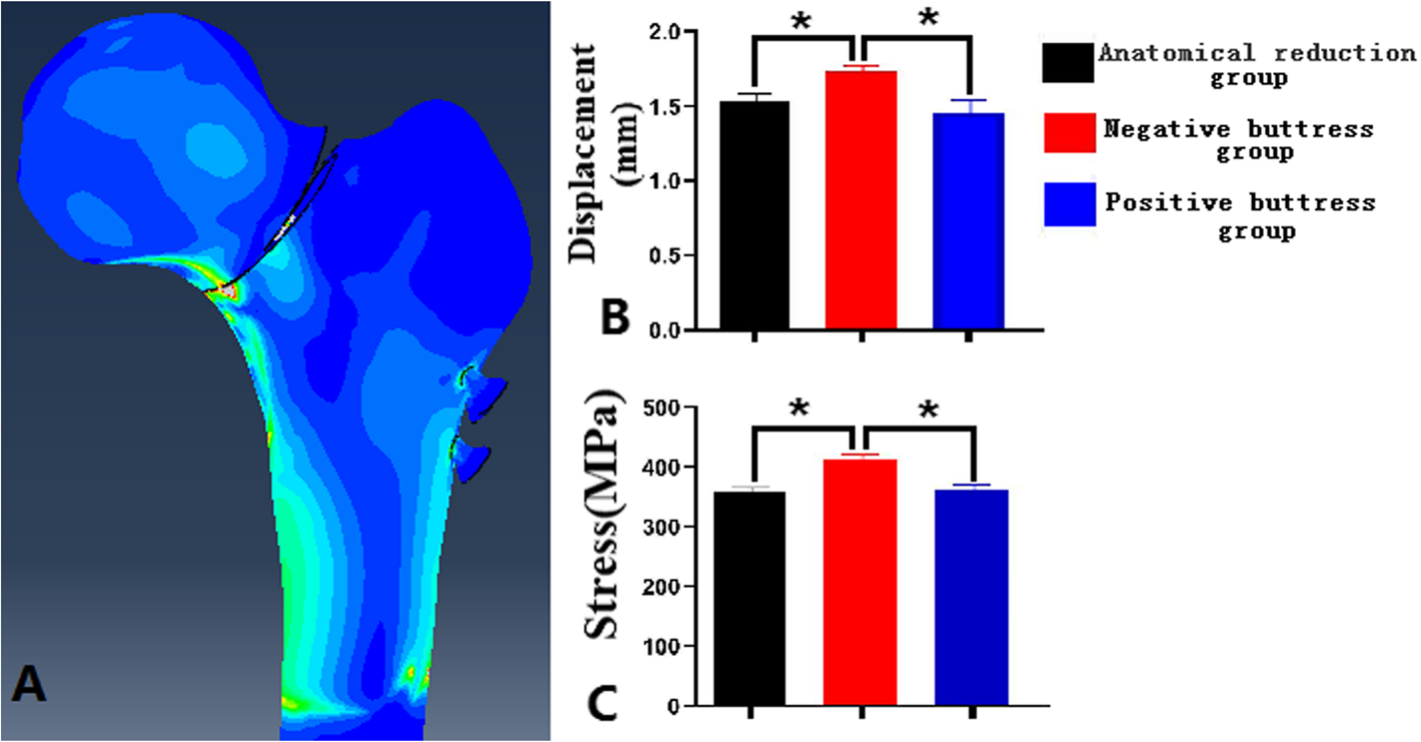
Comparison of stress and displacement among three groups, **A** stress cloud; **B** displacement; **C** stress (*P < 0.05)

The finite element stress map showed that the mechanical conduction of the femur could be effectively restored after internal fixation, with the greatest screw stress, followed by the femoral neck site. There was significant difference in the average displacement of fracture end among the three groups (P < 0.05). In addition, the mean stress of the calcar also showed corresponding results, which was greater in the negative buttress group and the positive buttress group (P < 0.05, Fig. 3).

### Typical case

#### Typical case 1: Anatomical reduction

**Fig. 4:**
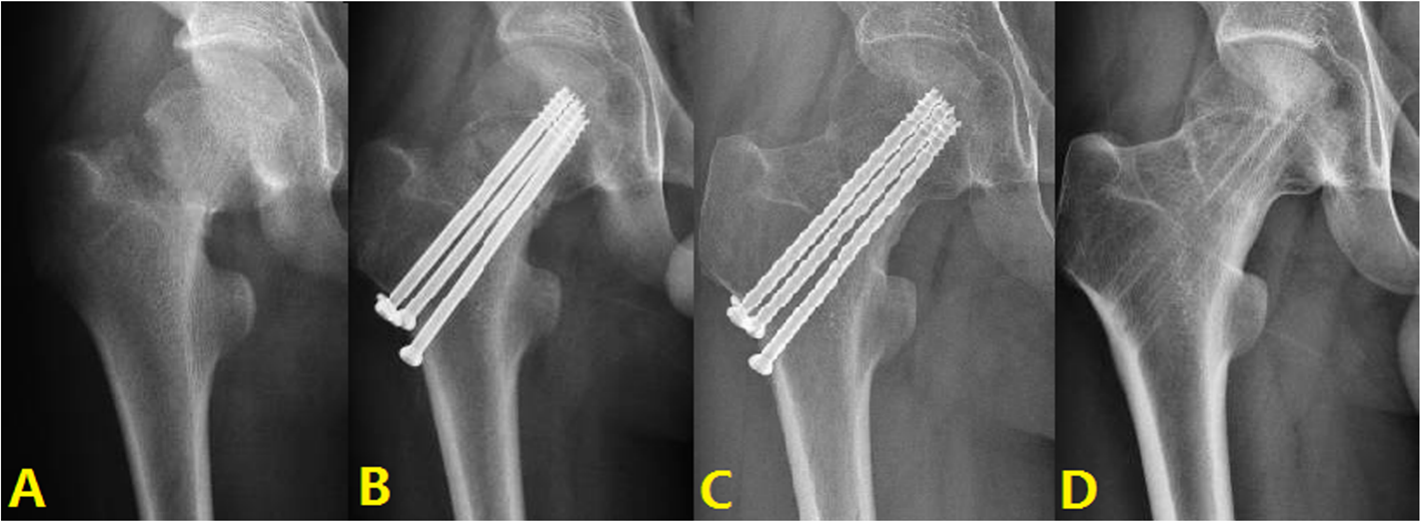
Typical cases of anatomical reduction: **A** Fracture of right femoral neck. **B** Screw internal fixation. **C** 1 year after surgery. **D** 3 years after surgery

A 43-year-old male presented with right hip pain with limited mobility due to a crash. The patient received three cannulated screws surgery. The fracture was anatomically reduced. Satisfactory results after 3 years of follow-up (Fig. 4).

#### Typical case 2: Negative buttress

**Fig. 5:**
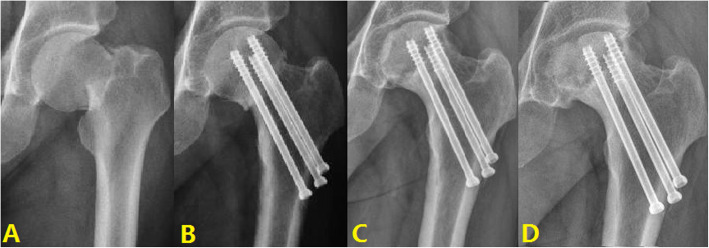
Typical cases of negative buttress: **A** Fracture of left femoral neck. **B** Screw internal fixation. **C** 2 year after surgery. **D** 4 years after surgery

A 51-year-old male presented with left hip pain with limited mobility due to a crash. A diagnosis of left femoral neck fracture was made, and after excluding surgical contraindications, closed reduction and cannulated screw internal fixation of the left femoral neck fracture was performed. The first postoperative reexamination showed negative buttress, and the patient was followed up for 4 years after operation, with femoral head necrosis (Fig. 5).

#### Typical case 3: Positive buttress

**Fig. 6:**
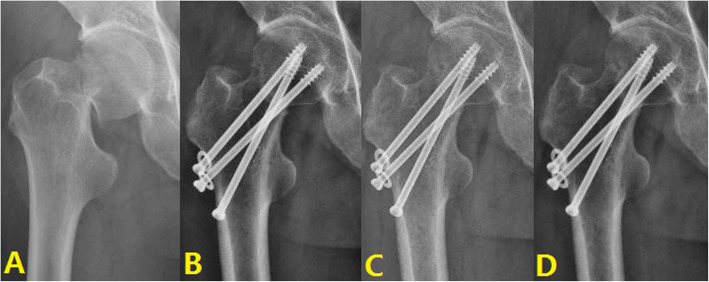
Typical cases of positive buttress: **A** Fracture of right femoral neck. **B** Screw internal fixation. **C** Half a year after surgery. **D** 2 years after surgery

A 42-year-old man with pain in the right hip joint and limited mobility due to a crash was diagnosed with a right femoral neck fracture and underwent closed reduction and internal fixation with cannulated screws for a right femoral neck fracture after surgical contraindications were excluded. The first postoperative film reexamination showed positive buttress, and the fracture healed well and no complications were observed during the 2-year postoperative follow-up (Fig. 6).

## Discussion

The treatment of femoral neck fractures in young- and middle-aged adults has been highly controversial. The quality of fracture reduction is essential for fracture healing and avoiding postoperative complications. In this study, through clinical retrospective analysis and finite element analysis, it was found that both anatomical reduction and positive buttress could achieve better clinical results, and postoperative complications such as femoral head necrosis, femoral neck nonunion, and femoral neck shortening were better than the negative buttress group.

Fracture reduction can affect the femoral head, any adverse reduction (femoral head rotation, varus and valgus or poor reduction of the fracture end, etc.) may increase the incidence of avascular necrosis of the femoral head or femoral head collapse [18, 19]. Anatomic reduction can better protect the femoral head, but if anatomical reduction is excessively pursued, especially in the treatment of irreducible femoral neck fracture, it is easy to cause repeated traction and rotation, which will destroy the blood supply of the femoral bone and increase the risk of avascular necrosis of the femoral head and nonunion [20, 21]. Yechiel Gotfried found through clinical experience that negative buttress was associated with a high failure rate of the procedure and advocated valgus and positive buttress reduction. The positive buttress reduction can be performed by the Yechiel Gotfried reduction method it summarizes. By using the YechielGotfried reduction method, he treated 18 cases of subcapital femoral neck fractures, of which 5 cases were followed up for at least 1 year without complications such as fracture redisplacement, nonunion, or femoral head necrosis [13]. Although the Yechiel Gotfried reduction theory is very promising, the theory needs to be confirmed by a large number of clinical case analyses and follow-up. The clinical statistics of this study found that the Harris scores of the three groups increased in turn at 3 months, 6 months, and 12 months after operation. At 6 months and 12 months after operation, the negative buttress component had the lowest value, the hip joint function was worse than the other two groups, and there was no significant difference in the hip joint function during postoperative follow-up in the positive buttress group and anatomical reduction group. Femoral neck fracture internal fixation mostly uses sliding design [22]. Under the action of the patient’s own weight, the femoral head can slide to the base of femoral neck along the long axis of internal fixation, thus playing a role in compression fixation and promoting fracture healing [23]. However, the ability of the design to resist shear force is weak. If it moves too early, it is easy to cause internal fixation breakage and bone block cutting, prolong the time of bed rest, but it is not conducive to fracture healing [24]. As the medial femoral neck cortex is significantly thickened and the calcar is strengthened medially and posteriorly, a bridge arch is formed on the medial side of the femoral neck [25]. When positive buttress reduction is achieved, due to the sliding compression of the femoral head to form impaction, the medial cortex distal to the fracture straddles on the medial femoral neck support bridge, and the special stress transfer effect of the arch structure can effectively resist the longitudinal shear force between the fracture blocks and stabilize the fracture [26]. This may be the reason why the hip joint function was good in patients with positive buttress.

In addition to hip function, postoperative complications are also an important concern for screw internal fixation in the treatment of femoral neck fractures [27]. Common postoperative complications of femoral neck fracture include osteonecrosis of the femoral head, delayed fracture healing, and femoral neck shortening. It has been reported in the literature that the incidence of postoperative complications after screw internal fixation for femoral neck fracture is inconsistent and related to many factors, such as orthopedic surgeons’ surgical experience, quality of reduction, time to initial weight bearing, patient bone metabolism, and rehabilitation exercises [28, 29]. In this study, the incidence of postoperative complications of femoral neck fracture was observed by clinical observation of different reduction conditions. In terms of postoperative femoral head necrosis, femoral neck shortening, and fracture nonunion, the negative buttress group was more likely to have it. The incidence of osteonecrosis was as high as 32.2%. And 27.4% of patients had femoral neck shortening. The complication rate of positive buttress and anatomical reduction was comparable and lower than that of negative buttress group. The incidence of femoral neck shortening was lower in the positive buttress group possibly due to the support of the medial cortical buttress bridge of the femoral neck below the fracture fragment. Therefore, for patients with positive buttress reduction and fracture dislocation within the acceptable range, anatomical reduction is no longer required.

From a biomechanical point of view, the maximum displacement value of the fractured end under the applied load can directly reflect the stability of the model, and the smaller the displacement value, the stronger the fixation [30]. This study showed that the fracture end displacement of the positive buttress group was smaller than that of the negative buttress group under the same load, that is, the stability was better. In addition, the object will produce a certain deformation under the action of external force, and the degree of deformation is called strain [31]. The mean stress of the calcar was greater in the negative group than in the positive group, showed that positive reduction or anatomical reduction can well restore normal stress conduction in the hip joint. Despite the above findings, the present study has the following limitations. First, the clinical part of this study is a retrospective analysis study, with a limited number of cases, which may have selection bias; second, the finite element part of this study simulates the model to simplify cartilage, muscle attachment, etc. Although finite Element Software tools do not replace experimental testing, they provide a valuable and rapidly evolving option [32]. Furthermore, in this study, only Pauwels type III femoral neck fractures were analyzed by finite element analysis. Our future work is to design a large sample of research to confirm the advantages of positive buttress.

## Conclusion

Both positive buttress and anatomical reduction in the treatment of femoral neck fracture with cannulated screw internal fixation can obtain better clinical effect and lower postoperative complications. Positive brace support and anatomic reduction can limit the restoration of femoral stress conduction. The negative buttress reduction should be avoided.

## Data Availability

All the data will be available upon motivated request to the corresponding author of the present paper.

## Abbreviations

CT: Computed tomography
MRI: Magnetic resonance imaging
DICOM: Digital imaging and communications in medicine

## References

[1] Rogmark C, Leonardsson O. Hip arthroplasty for the treatment of displaced fractures of the femoral neck in elderly patients. Bone Joint J. 2016;23:291–7. 10.1302/0301-620X.98B3.36515 26920951

[2] Young-Hoo K, Young-Soo J (2021). Long-term clinical and radiographic results of an ultra-short metaphyseal-fitting non-anatomic cementless stem in patients with femoral neck fracture. J Arthroplasty.

[3] Wang T, Jun-Ying S, Guo-Chun Z (2014). Analysis of risk factors for femoral head necrosis after internal fixation in femoral neck fractures. Orthopedics.

[4] Wan L, Xiangyun Z, Wu D (2021). Application of robot positioning for cannulated screw internal fixation in the treatment of femoral neck fracture: retrospective study. JMIR Med Inform.

[5] Yipeng W, Muguo S, Guangliang P (2021). Muscle pedicle bone flap transplantation for treating femoral neck fracture in adults: a systematic review. J Orthop Surg Res.

[6] Pengfei X, Yonggang T, Zhinan H (2020). The clinical and radiographic characteristics of avascular necrosis after pediatric femoral neck fracture: a systematic review and retrospective study of 115 patients. J Orthop Surg Res.

[7] Fang P, Rui Z, Fenglei L (2020). Osteonecrosis of femoral head in young patients with femoral neck fracture: a retrospective study of 250 patients followed for average of 7.5 years. J Orthop Surg Res.

[8] Combalia M, Muñoz-Mahamud E, Febles-Oviedo JL, et al. Spontaneous non-traumatic dislocation of the hip as a complication of screw-plate fixation of a femoral neck fracture. Injury. 2021;22:326. 10.1016/j.injury.2021.02.063 33736861

[9] Lund Erik A, Rahul S, Mark W (2020). Association of perioperative computed tomography Hounsfield units and failure of femoral neck fracture fixation. J Orthop Trauma.

[10] Ta-Wei T, Fang-Chien L, Pei-Yuan L, et al. Using a cannulated screw as a drill guide and sleeve: a simple technique for multiple-screw fixation for intracapsular femoral neck fracture. Orthopedics. 2010;33(8). 10.3928/01477447-20100625-05 20704114

[11] Yanling S, Wei C, Qi Z (2011). An irreducible variant of femoral neck fracture: a minimally traumatic reduction technique. Injury.

[12] Wang Z, Luosha G, Cheng L (2020). Open reduction and internal fixation and intraoperative exploration of the superior retinacular arterial system in young adults Garden III femoral neck fracture: a 10 case report. Ann Plast Surg.

[13] Yechiel G, Sergey K, Dror F (2013). Nonanatomical reduction of displaced subcapital femoral fractures (Gotfried reduction). J Orthop Trauma.

[14] Kai H, Xiaohui F, Guijun L (2020). Assessing the effect of Gotfried reduction with positive buttress pattern in the young femoral neck fracture. J Orthop Surg Res.

[15] Mediouni M, Schlatterer DR (2017). Orthopaedic tumors: what problems are we solving, and are universities and major medical centers doing enough?. J Orthop..

[16] Mediouni M, Schlatterer DR, Khoury A, Von Bergen T, Shetty SH, Arora M, Dhond A, Vaughan N, Volosnikov A. Optimal parameters to avoid thermal necrosis during bone drilling: A finite element analysis. J Orthop Res. 2017;35(11):2386–91. 10.1002/jor.23542 28181707

[17] Lin T, Yang P, Xu J, et al. Finite element analysis of different internal fixation methods for the treatment of Pauwels type III femoral neck fracture. Biomed Pharmacother. 2019;112:108–658. 10.1016/j.biopha.2019.108658 30970508

[18] Xu J-L, Zheng-Rong L, Bing-Lang X (2019). Risk factors associated with osteonecrosis of femoral head after internal fixation of femoral neck fracture: a systematic review and meta-analysis. BMC Musculoskelet Disord.

[19] Roger E, Marion S, Guillaume V (2020). Poor results of functional treatment of Garden-1 femoral neck fracture in dependent patients. Orthop Traumatol Surg Res.

[20] Chingiz A, Afgan J, Farhad A (2020). Efficiency of an implant: new criterion of objective assessment of implants for osteosynthesis of femoral neck fracture. Int Orthop.

[21] Jia L, Pengbin Y, Licheng Z (2019). Medial anatomical buttress plate in treating displaced femoral neck fracture a finite element analysis. Injury.

[22] Shibo H, Benjie W, Xiuzhi Z (2020). High-purity weight-bearing magnesium screw: translational application in the healing of femoral neck fracture. Biomaterials.

[23] Fa-Chuan K, Kai-Lan H, Lin C-L (2019). Biomechanical properties of off-axis screw in Pauwels III femoral neck fracture fixation: bicortical screw construct is superior to unicortical screw construct. Injury.

[24] Jingwen L, Baokun Z, Bohao Y (2019). Biomechanical evaluation of the modified cannulated screws fixation of unstable femoral neck fracture with comminuted posteromedial cortex. Biomed Res Int.

[25] Pontus S, Volker O, Olof W (2019). Posterior and anterior tilt increases the risk of failure after internal fixation of Garden I and II femoral neck fracture. Acta Orthop.

[26] Wang G, Wang B, Wu X (2020). Gotfried positive reduction promotes the repair of femoral neck fracture potentially via enhancing osteogenesis and angiogenesis. Biomed Pharmacother.

[27] Ugland TO, Haugeberg G, Svenningsen S, et al. High risk of positive Trendelenburg test after using the direct lateral approach to the hip compared with the anterolateral approach: a single-centre, randomized trial in patients with femoral neck fracture. Bone Joint J. 2019;18:793–9. 10.1302/0301-620X.101B7.BJJ-2019-0035.R1 PMC6617057 31256660

[28] Wu X-B, Jun-Qiang W, Xu S (2019). Guidance for the treatment of femoral neck fracture with precise minimally invasive internal fixation based on the orthopaedic surgery robot positioning system. Orthop Surg.

[29] Orlin F, Karl S, Boyko G (2017). Femoral neck fracture osteosynthesis by the biplane double-supported screw fixation method (BDSF) reduces the risk of fixation failure: clinical outcomes in 207 patients. Arch Orthop Trauma Surg.

[30] Shabnam S, Peter A, Gholamreza R (2019). Stability of femoral neck fracture fixation: a finite element analysis. Proc Inst Mech Eng H.

[31] Lu H, Hongquan S, Shuqing Z (2020). Biomechanical analysis of the computer-assisted internal fixation of a femoral neck fracture. Genes Dis.

[32] Mediouni M, Kucklick T, Poncet S (2019). An overview of thermal necrosis: present and future. Curr Med Res Opin..

